# A Probabilistic Analysis of Sparse Coded Feature Pooling and Its Application for Image Retrieval

**DOI:** 10.1371/journal.pone.0131721

**Published:** 2015-07-01

**Authors:** Yunchao Zhang, Jing Chen, Xiujie Huang, Yongtian Wang

**Affiliations:** 1 School of optoelectronics, Beijing Institute of Technology, Beijing, China; 2 School of computer science & technology, Beijing Institute of Technology, Beijing, China; Xiamen University, CHINA

## Abstract

Feature coding and pooling as a key component of image retrieval have been widely studied over the past several years. Recently sparse coding with max-pooling is regarded as the state-of-the-art for image classification. However there is no comprehensive study concerning the application of sparse coding for image retrieval. In this paper, we first analyze the effects of different sampling strategies for image retrieval, then we discuss feature pooling strategies on image retrieval performance with a probabilistic explanation in the context of sparse coding framework, and propose a modified sum pooling procedure which can improve the retrieval accuracy significantly. Further we apply sparse coding method to aggregate multiple types of features for large-scale image retrieval. Extensive experiments on commonly-used evaluation datasets demonstrate that our final compact image representation improves the retrieval accuracy significantly.

## Introduction

Most state-of-the-art image retrieval approaches rely on bag-of-words (BoW) framework and its variants [1–3] based on local descriptors. Although the BoW model makes it possible to be used for image quantization and the TF-IDF inverted indexing structure originated from web text search are applied to find the closest image in the database, followed by a re-ranking of the result list based on geometric considerations. However it suffers from visual word ambiguity, feature quantization error and memory constraints.

Another promising image retrieval approach is proposed by aggregating local descriptors on one image into a compact vector using fisher vector (FV) [4] or Vector of Local Aggregated Descriptor (VLAD) [5,6]. Compared with BoW model, FV and VLAD vector is formed from visual words residuals while not the frequency of words. Fisher vector and VLAD are memory-demanding, which need to use compression methods to make them tractable for large-scale applications. But the need for decompression before retrieval reduces the efficiency. Furthermore, large-scale feature k-means clustering is still necessary to generate a compact codebook, this clustering process will be time-consuming, which will take several hours even only a few database images are updated or added additionally.

Recently sparse coding has been widely used in object recognition [7], image classification [8], image denoising [9] and image inpainting [10]. By using sparse coding instead of vector quantization, each feature extracted from one image can be represented by a high dimensional but sparse and fixed length vector. Therefore sparse coding has been suggested as a promising method for approximate nearest neighbor (ANN) in the recent past [11–14]. The work in [8] indicates that sparse coding with max-pooling that chooses the largest coefficient for a visual word can outperform the performance of the state-of-the-art image retrieval. While promising, a major difficulty affecting the performance of these methods is that the sparse codes generated by learned dictionaries are often found to be seriously affected by many factors, such as sparsity, feature sampling and pooling strategies, which are still poorly understood. In view of the above problems, we make three contributions in this research. Our first contribution is that we give a probabilistic interpretation to the max or sum pooling operation in the context of sparse coding framework. Motivated by this probabilistic interpretation, we explain the relationship of feature extraction and pooling strategies. Second, we propose a modified sum pooling procedure which can improve the retrieval accuracy significantly, especially for smaller visual vocabularies. Finally our third contribution is that we exploit sparse coding method to aggregate multiple types of features for large-scale image retrieval. Extensive tests with several state-of-the-art descriptors have been performed and gained excellent results.

The rest of this paper is organized as the following: Section 2 gives the related work and the background of aggregating local features. Section 3 we analyze the effects of pooling strategies with a probabilistic explanation in the context of sparse coding framework and propose a new modified sum pooling method. Aggregating multiple features in using sparse coding framework with our pooling method is described in Section 4. Finally the experimental results and conclusion are given in Section 5 and 6.

## Related Work

### Sampling Schemes

Feature sampling is the first step of many vision applications, such as image classification and image retrieval. A great deal of work has focused on feature sampling schemes [15, 16]. Sparse sampling and dense sampling are two popular feature sampling strategies. Dense sampling is commonly used in image classification while sparse sampling is usually adopted for image retrieval applications.

Dense sampling scheme can obtain a large number of patches uniformly sampled with a fixed step. In the reference [17], the authors are the first to verify the effectiveness of dense sampling for image classification. However, those dense patches not only provide a better coverage of interesting objects but also a lot of redundant information such as the blue sky and the clean ground. Information contained in these patches maybe greatly repeated and can be regarded as the burstness phenomenon [18]. The burstness has a greater impact on sum pooling operation compared with max pooling.

Key-point based sparse sampling aims at extracting distinctive and repeatable features in an image. SIFT [19], SURF [20] and Hessian-Affine detector [21] are traditional sparse feature sampling methods. These sampled patches are not uniformly distributed and may be crowded in some background regions. The scattered clutters in the background may affect a lot for the max pooling operation. We will give the theoretical and experimental explanation for the selection of sampling schemes in section 3 and 5.

### Coding Schemes

#### Bag of Words

Traditionally, in the BoW model, the vector quantization (VQ) is applied to encode the local descriptors into discrete visual words, which has been proved to be simple and efficient in dealing with the problem of large-scale image retrieval. To reduce the visual word ambiguity, Hamming embedding, weak geometry consistence [22] and soft assignment [23] are proposed to improve the discriminative power of local features in a bag of words framework. Meanwhile, codebook compression method [24] is proposed to deal with the high-dimensional bag of words histogram while maintaining its visual discriminability. The authors in [25] designed the Spatial-Bag-of-Features by projecting the image features to different directions or points to generate a series of ordered BOF, then selecting the most representative features to generate a new BOF-like vector representation of an image. In [26], the authors proposed to use bag of hash bits instead of bag of words to do mobile visual search. Each local feature is encoded to bag of hash bits by using similarity preserved hashing functions such as PCA hashing or SPICA hashing [27]. In order to improve retrieval efficiency, the authors in [28] used heading information from digital compass to facilitate the BOF descriptors generation process.

#### Residual Vector Quantization

Another more scalable vector quantization method applied for image retrieval was achieved with the compressed Fisher vector [4] and VLAD [5]. Database image representations are also generated from local descriptors like SIFT or SURF, yet they utilized an alternative aggregation stage to replace bag-of-words histograms. For compressed fisher vector method mentioned in reference [4], the codebook is generated by using a Gaussian mixture model with *K* components {(*ω*_*i*_,*u*_*i*_,Σ_*i*_),*i* = 1,2,…*k*}, where *ω*_*i*_,*u*_*i*_,Σ_*i*_ are the weight, mean and covariance of the *i*-th Gaussian model learned on offline stage using Maximum Likelihood method. The gradient vector for a local descriptor *x*_*i*_ is represented as:

$$v(x_{i})=[\xi_{1},\xi_{2},\ldots ,\xi_{N}],\xi_{i}=\frac{1}{\sqrt{\omega }}\gamma (i)\sigma_{i}^{-1}(x-u_{i})$$ (1)

Here $\gamma (i)=\omega_{i}p_{i}/\sum_{j=1}^{N}\omega_{i}p_{j}$ is the probability of descriptor belonging to the *i*-th Gaussian model. As the authors mentioned in their work [5], VLAD is a simplified non-probabilistic version of the fisher vector. For VLAD, each descriptor *x*_*i*_ is associated to its nearest visual word *δ*(*c*_*k*_ = *NN*(*x*_*i*_)) to generate the vector *v*(*x*_*i*_) = [0,…,*x*_*i*_ − *c*_*k*_,…,0]. Then the image representation vector *V* can be obtained by concatenating the aggregated residual vector *v*(*x*_*i*_).

Based on VLAD approach, the Residual Enhanced Visual Vector (REVV) [29] is developed to further reduce the database’s memory usage. Besides, LDA is employed for dimension reduction and several features like SURF and CHoG [30] are used together to improve the retrieval accuracy. Considering the projection errors generated in the dimension reduction process, which may inevitably decrease the search accuracy, the authors in [31] proposed a method of projected residual vector quantization.

#### Sparse Coding

Given an input signal *x*, the sparse coding seeks to reconstruct *x* using a linear combination of an over-complete dictionary *C* with a sparse coefficient vector *v*. The generative model for representing an input signal *x* can be written as:

$$\underset{V}{\min}{\left\|x-Cv\right\|}_{2}^{2}+\lambda {\left\|v\right\|}_{1},s.t.v>0$$ (2)

Where *λ* is the parameter to control the sparsity of *v*. Amount of research have been done to solve the Eq (2) with the *L*_1_-norm, such as Lasso [32] and feature-sign search algorithm [33]. As we can see from Eq (2), for bag of words approach the vector not only need to satisfy *v* > 0, but also is restricted by *card*(*d*) = 1 and |*v*|_0_ = 1. The constraint |*v*|_0_ = 1 is relaxed to |*v*|_0_ = *n* for soft assignment. While for sparse coding, these constraints are relaxed by putting *L*_1_ norm regularization on which can give a more accurate reconstruction of *x*. Therefore to some extent, the BoW frequency histogram is an approximation of sparse coding.

In sparse coding framework, several factors affect the retrieval accuracy, for example, feature extraction, dictionary learning, and feature pooling. In [11], the authors proposed a novel scheme of dictionary learning for sparse coding. In reference [34], the authors decomposed sparse coding problem into smaller subproblems, the codebook is a Cartesian product of two subcodebooks, which improved the retrieval speed significantly. In [35], the authors theoretically analyzed the max/sum pooling scheme and the effects of pooling cardinality for image classification. We extend the work in reference [35] to analyze several other factors that affect pooling performances and apply it to retrieval works. Furthermore, we propose a modified pooling strategy.

### Pooling Schemes

Given the sparse coefficients of all descriptors in an image, a pooling operation is often used to obtain an image level representation vector. Sum-pooling, average-pooling and max pooling are the popular pooling methods used for image retrievals. On pooling stage, BoW, FV and VLAD calculate the sum of the vector *v*(*x*_*i*_) to aggregate all encoded vector into a single vector. While for REVV, median pooling scheme is used to aggregate local features. However, sparse coding with max-pooling has demonstrated its higher classification performance than sum-pooling and average-pooling with dense sampling strategies [36–38]. Furthermore, a new mix-order max-pooling operation, which incorporates the probability and the frequency of the presence of a visual word in an image, is proposed to obtain a more informative image-level representation further [39]. Thus, some experimental results show that sparse coding with max pooling can achieve better performance for large scale image retrieval [14].

Aggregating of multiple features is often another way to be used to improve the retrieval performance, as single feature may miss some information of original image. In [40–42], the authors proposed that commonly used features for each image can be divided into three different levels, which are low-level, mid-level and high-level features. These features can be mutual complementation for image retrieval if well combined. Low-level features are those features directly extracted from the original images, such as SIFT, SURF, color and many other pixel level features. Recently, many successful researches for image retrieval transform multiple low-level features into a global image representation. The authors in [43] combined SIFT and GIST by graph fusion and maximizing weighted density for accurate image retrieval. The authors in [44] proposed a coupled Multi-Index framework to perform feature fusion for image retrieval, in which SIFT and color features are combined. Furthermore, based on the research of aggregating multiple low-level features, the authors in [40] proposed multi-graph learning method to explore the complementation of different level features, which can be used for specific field, such as social image retrieval.

## Sampling and Pooling Strategies under a Sparse Coding framework

Obviously, sparse coding with different sampling and pooling schemes can dramatically affect the classification and retrieval performance. However, the reasons to select dense or sparse sampling for image retrieval and the mechanism of max and sum pooling schemes under a sparse coding framework for image retrieval have not been deeply understood yet. Therefore in this paper, we first give a theoretical analysis of which sampling strategy to select, and then we provide some probabilistic explanations to the max or average pooling operation. Based on the probabilistic explanations we propose a modified pooling strategy applied for image retrieval in the context of sparse coding framework.

### Selection of Sampling Strategies

In this paper, our work is focused on the sparse coding for image retrieval. Although dense sampling scheme is widely used in classification works, it is not deemed to work so well in image retrieval. As image retrieval is an unsupervised learning processing in most cases, which we cannot exclude the repeated and redundant information as the learned classifier does in image classification. However, we found that it is not always true. Dense sampling outperforms sparse sampling in some cases.

**Fig 1:**
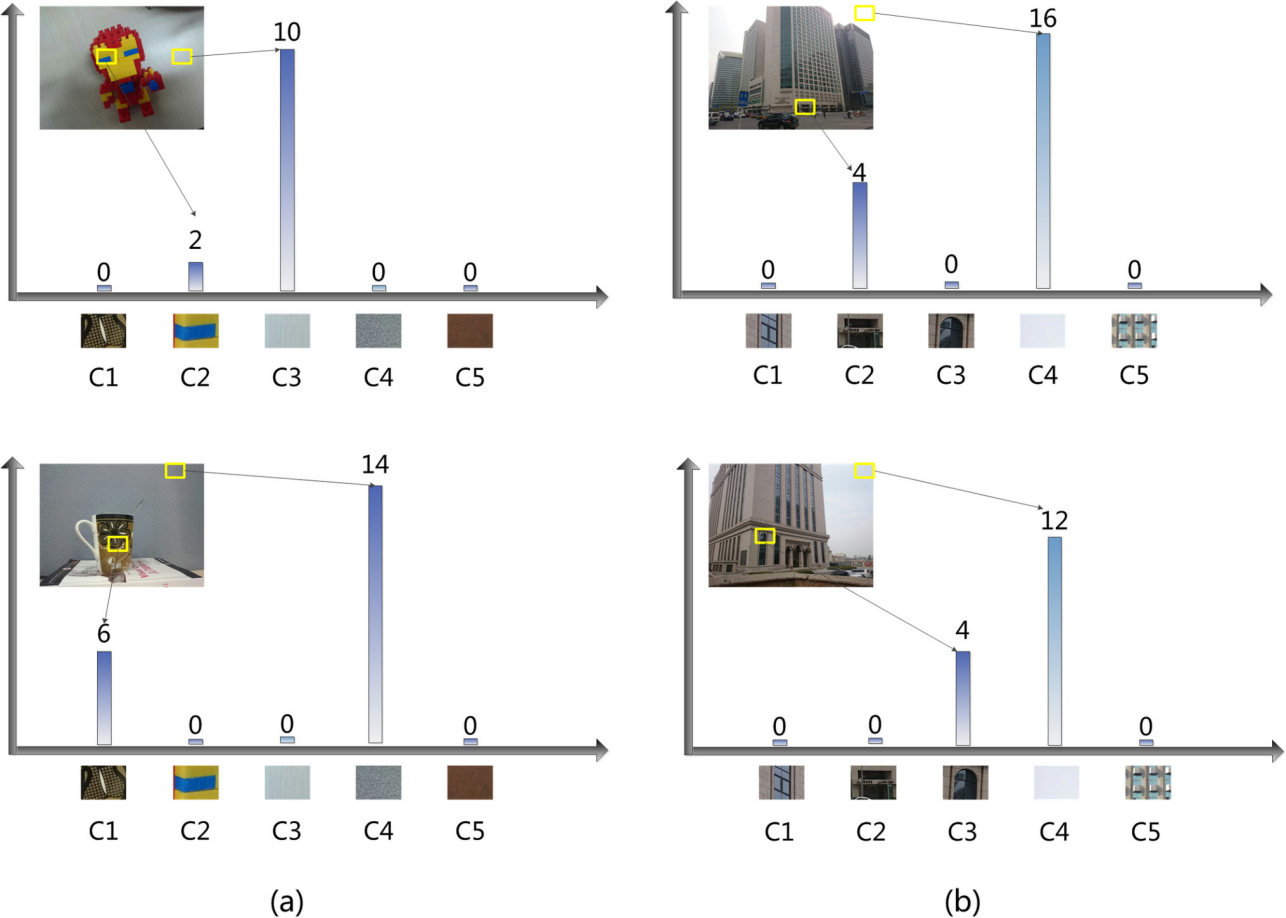
Histogram of sum pooling results. (a) Visual codes C3, C4 generated by distinctive patches on the background. (b) Visual codes C4 generated by frequent patches on the background.

Dense sampling extracts the patches uniformly which may contain lots of repeated and redundant information on the clean background. The repeated patches can be divided into two categories: 1) distinctive patches are denoted as those repeated patches which are present in a little part of train images and 2) frequent patches are denoted as those repeated patches which are present in most of the training images. Fig 1 illustrates the feature coding step of dataset images. Dense patches of training images are extracted and a codebook with five visual codes is trained. The sum pooling results of images on the codebook generate a histogram. The repeated patches on the left images fall into various bins. The visual code C3 and C4 have a strong discrimination. However, the repeated patches on the right images fall into the same bin. The code C4 has low discrimination. It is obviously to be seen that the distinctive patches on the background can contribute to improving the retrieval performance while frequent patches will not. It is similar to some extent with thought of IDF in BOW model, which is not included in sparse coding framework.

Sparse sampling can be regarded as a special case of dense sampling which excludes some repeated features in some clean regions. It is a subset of dense sampling. From above analysis, we can easily get that the selection of sampling method is greatly affected by the dataset we choose. Dense sampling may work well with distinctive patches for image retrieval, and sometimes outperform sparse sampling. While, dense sampling will achieve bad retrieval performance with frequent patches, especially when sum pooling is used. It is also indicated in Section 5.1.

### Probabilistic Interpretation of Sum and Max Pooling Operations

In sparse coding representation, sparse coefficients often follow exponential distributions as mentioned in [35, 37,39, 45–47]. The coding of a single feature can be regard as a random experiment *χ*_*i*_ on code words. Assume the sparse coefficient of feature *x*_*i*_ on a visual word *j* is *α*_*i*,*j*_, *N* features coded on the visual word *j* can generate a series of independent random variables. Suppose *α*_1,*j*_,*α*_2,*j*_, …, *α*_*N*,*j*_ are *N* mutually independent random variables having exponential distribution with the parameter *λ*, and they are assumed to be independent identically distributed (IID) random samples with density *f*(*α*) = *λ*exp^−*λα*^ and cumulative distribution function *F*(*α*) = 1 − exp^−*λα*^. The expectation of sparse coefficients *α* are $\mu =\frac{1}{\lambda }$ and $\sigma =\frac{1}{\lambda^{2}}$ are the variance. Pooling steps of *N* features can be modeled as a combination of such random variables. Although the probabilistic interpretation described here is similar with the work in reference [35], we supply the derivation process of probability distribution for max/sum pooling and extend the probabilistic explanations. Furthermore, we introduce more factors which may affect image retrieval performance, such as the number of code words.

#### Max pooling

Max pooling selects the maximum value of *N* random experiments on a visual word *j* as the pooling result.

$$\alpha_{\max,\text{j}}=\max(\alpha_{1,j},\alpha_{2,j},\ldots ,\alpha_{N,j})$$ (3)

The corresponding joint probability distribution function of max pooling can be written as:

$$F_{\max}(\alpha )=P(\alpha_{1,j}<\alpha ,\alpha_{2,j}<\alpha ,\ldots ,\alpha_{N,j}<\alpha )=\prod_{i=1}^{N}P(\alpha_{\text{i},j}<\alpha )={\left(1-\exp^{-\lambda \alpha }\right)}^{N}$$ (4)

The expectation of joint probability distribution is:

$$E_{\max}(\max(\alpha_{1,j},\alpha_{2,j},\ldots ,\alpha_{N,j}))=\mu \sum_{i=1}^{N}\frac{1}{i}\approx \mu (l+\log N)$$ (5)

Here l is Euler's constant. And the variance of joint probability distribution is:

$$D_{\max}(\max(\alpha_{1,j},\alpha_{2,j},\ldots ,\alpha_{N,j}))=\sigma^{2}\sum_{i=1}^{N}\frac{1}{i^{2}}<2\sigma^{2}$$ (6)

#### Sum/Average pooling

Sum/average pooling selects the sum or average value of *N* random experiments on a visual word *j* as the pooling result, which can be represented as:

$$\alpha_{sum,j}=\sum_{i=1}^{N}\alpha_{i,j},\;\alpha_{avg,j}=\frac{1}{N}\sum_{i=1}^{N}\alpha_{i,j}$$ (7)

The probability density function of sum pooling and average pooling will be as following:

$$f_{sum}(\alpha )=\frac{\lambda^{N}\alpha^{N-1}\exp(-\lambda \alpha )}{(N-1)!}$$ (8)

$$f_{avg}(\alpha )=\frac{\lambda^{N}\alpha^{N-1}\exp(\frac{-\lambda \alpha }{N})}{N^{N-1}(N-1)!}$$ (9)

According to Lindburg-Levy central limit theorem, the corresponding joint probability distribution function of average pooling is approximate to Gaussian distribution when *N* is very big. Here *N* is the number of features which participate in the pooling. That means the Eq (9) can be rewritten as:

$$F_{avg}(\alpha )\approx \frac{1}{\sigma \sqrt{2\pi }}\int_{-\infty }^{\alpha }\exp(-\frac{{\left(t-u\right)}^{2}}{2\sigma^{2}})dt$$ (10)

The expectation of joint probability distribution can be written as:

$$E_{avg}(\frac{1}{N}\sum_{i=1}^{N}\alpha_{i,j})=\frac{1}{N}\sum_{i=1}^{N}\text{E}(\alpha_{i,j})=\mu$$ (11)

The variance of joint probability distribution will be:

$$D_{avg}(\frac{1}{N}\sum_{i=1}^{N}\alpha_{i,j})=\frac{1}{N^{2}}\sum_{i=1}^{N}D(\alpha_{i,j})=\frac{1}{N}\sigma^{2}$$ (12)

The similarity between query image and train images is commonly measured by the pooling vectors of encoded features. With good pooling strategy, we can easily separate similar images from dissimilar images. We employ *L*_1_ norm of pooling vectors $\sum_{i=1}^{k}\left\|\alpha_{i}^{q}-\alpha_{i}^{train}\right\|$ as the distance metric between query image and train images. As is well-known, the expectation of statistics can reflect the distribution information of them, so the expectation *E* of the sparse coefficients *α* can be used for the analysis of similarity measurement. According to the derived Eqs (5) and (11), the *L*_1_ norm of expectation $\sum_{i=1}^{k}\left\|E_{i}^{q}-E_{i}^{train}\right\|$ between query image and train images is $\sum_{i=1}^{k}\left(\mu_{i}^{q}-\mu_{i}^{train})(l+\log N\right)$ for max pooling and $\sum_{i=1}^{k}\left(\mu_{i}^{q}-\mu_{i}^{train}\right)$ for average pooling, here *N* is the pooling cardinality and *k* is the number of codewords.

**Fig 2:**
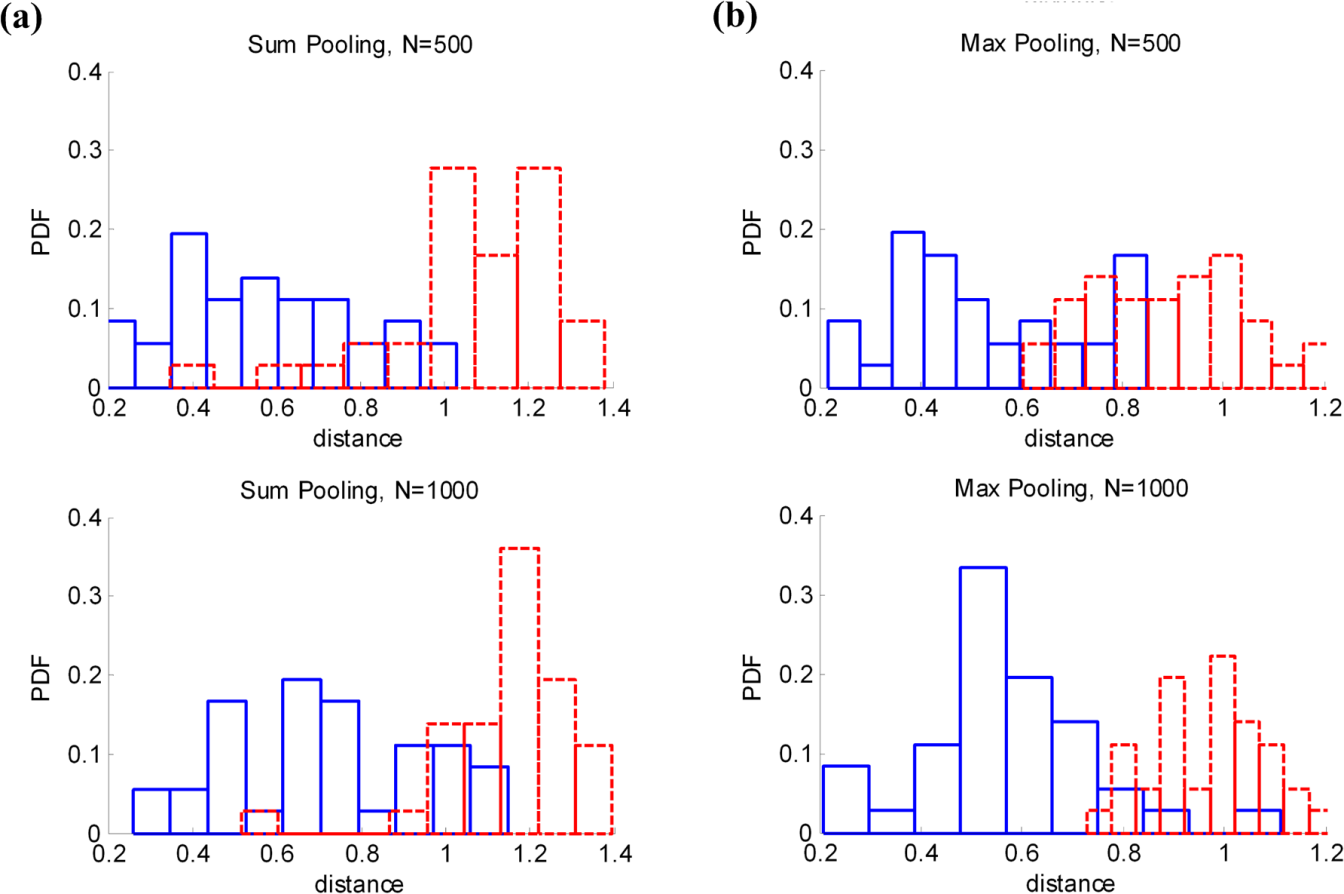
Distance between similar and dissimilar images on UKB dataset. (a) The probability density of *L*_1_ norm distance with sum pooling. (b) The probability density of *L*_1_ norm distance with max pooling.

As we can see from the *L*_1_ norm of expectation, max pooling tends to increase the discrimination of the similarity measurement than sum pooling, especially with the increasing of pooling cardinality *N*. Therefore similar and dissimilar images can be more easily separated with max pooling than sum pooling with the growth of pooling cardinality *N*. In order to proof this, we experimentally calculate the *L*_1_ norm distance $\sum_{i=1}^{k}\left\|\alpha_{i}^{q}-\alpha_{i}^{train}\right\|$ of similar and dissimilar images. Fig 2 shows the statistical frequency of the *L*_1_ norm distance with max and sum pooling schemes. The solid histogram stands for the probability density of *L*_1_ norm distance between similar images, while dashed histogram stands for dissimilar images. As shown in the statistical histogram in Fig 2, max pooling can easily separate similar images from dissimilar images with the increasing of pooling cardinality *N*. On the other hand, we can easily get that the retrieval performance of sum pooling and max pooling will both benefit from the growth of *k* codewords.

### A New Modified Sum Pooling Method

**Fig 3:**
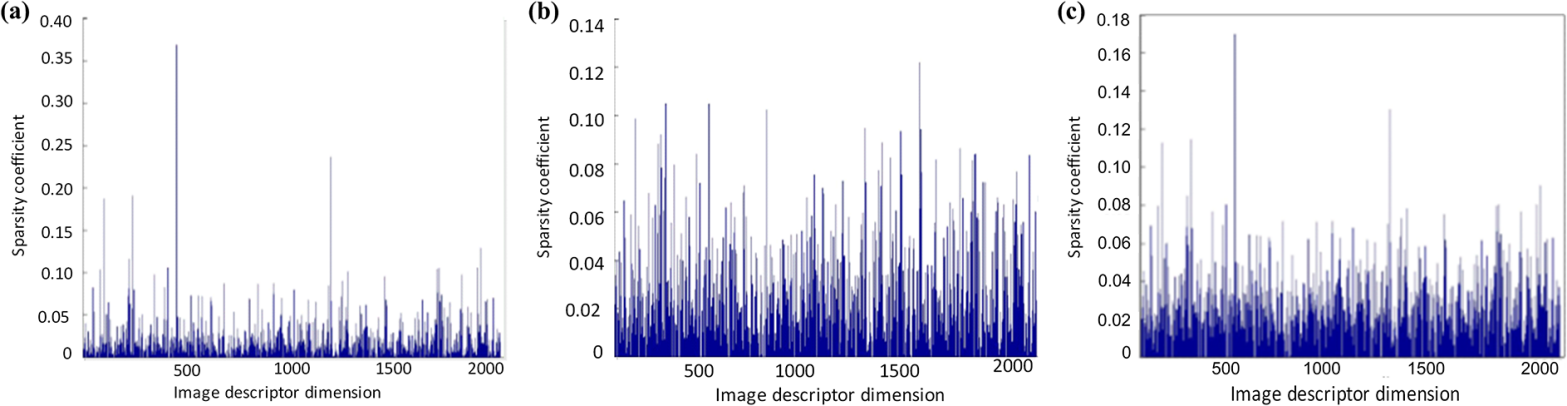
Impact on sparsity coefficient with different pooling schemes. (a) Sum pooling; (b) Max pooling; **(c)** Modified pooling.

However, retrieval performance can be influenced by a number of other factors, such as the burstness of features [18]. For sum pooling method, large amount of similar bursting features may have similar parameters on the same code word, which have a greater impact on $X_{avg}=\frac{1}{N}\sum_{i=1}^{N}X_{i}$ than on *X*_max_ = max(*X*_1_,*X*_2_,…,*X*_*N*_). Visual bursts would lead to some disruptive peak for average pooling, while max pooling is smoother. Fig 3 describes different pooling results of an image descriptor vector with a 2*K* dimensional codebook. Clearly, it can be observed that the value of a coding vector is strongly concentrated around only a few components with sum pooling (Fig 3A). These few components are responsible for a significant amount of energy and strongly influence the final query similarity scores, which lead to the contribution of other important dimensions decreased hugely. While with max pooling scheme this problem dose is alleviated, the large value is lower compared with sum pooling method. Obviously, max pooling strategy is prone to alleviate the higher weights of some visual words, which most probably are the bursty visual features.

Motivated by this observation, we propose a modified sum pooling method. In our pooling approach, each component of an image descriptor vector *v*_*i*_, *i* = 1,2,…,*k* is modified as *v*_*i*_ = |*v*_*i*_|^*β*^×*sign*(*v*_*i*_), *β*∈[0,1] to alleviate the strong influence caused by few components. Furthermore as shown in Fig 3A, there are some sparse coefficients which only have been assigned to a visual word once and the sparse coefficient is very small. Those small sparse coefficients are trivial and may be caused by computational errors.

Therefore in our pooling method we delimitate those sparse coefficients with Eq (13):

$$\alpha_{i}=0,\;\text{if}\;\alpha_{i}<Rank_{n}(\alpha ),\;\alpha_{i}={\left\{\alpha_{1},\alpha_{2},\ldots \alpha_{N}\right\}}^{T}.$$ (13)

Here *Rank*_*n*_(*α*) stands for the *n*-th largest sparse coefficients pooled in one code word. The top *n* scheme has better performance which has shown in reference [48]. Fig 3C shows a sparse coded vector pooled with our modified sum method.

## Sparse Coding with Multiple Features Using our Modified Pooling Method

Since sparse coding framework allows aggregating multiple types of features in a compact way, in this section we propose to apply sparse coding approach to combine multiple features to improve the retrieval performance further. We choose the popular SURF descriptor rather than SIFT, as consideration of memory and speed. SURF descriptor is a better choice than SIFT, especially for mobile landmark recognition [49–52]. Color information is a good complementary feature to SURF features. Because SURF features are extracted from the grey level images which do not contain any color information. We employ opponent color descriptor, which is more robust to illumination, scale and viewpoint change as mentioned in [53]. Around each key-point based detected SURF feature, we utilize a local patch with an area proportional to the scale of the key-point. Then a 36 dimension vector of this area is calculated as a color descriptor. For SURF and opponent color descriptors, codebooks are trained using conventional k-means method with independent SURF and color descriptors extracted from a set of real images. Every type of descriptors is quantized to the corresponding code words by feature-sign search algorithm [33] method to generate sparse coding signatures *α*. The final image representation vector will be the pooling results of those sparse coding signatures.

After sparse coding and feature pooling, all sparse coded multiple feature vectors are concatenated into a single one with different weights, which is different from [14], the combination is represented as following:

$$F=\left[\begin{array}{cc} \beta_{1}V_{sift} & \beta_{2}V_{color} \end{array}\right]$$ (14)

**Fig 4:**
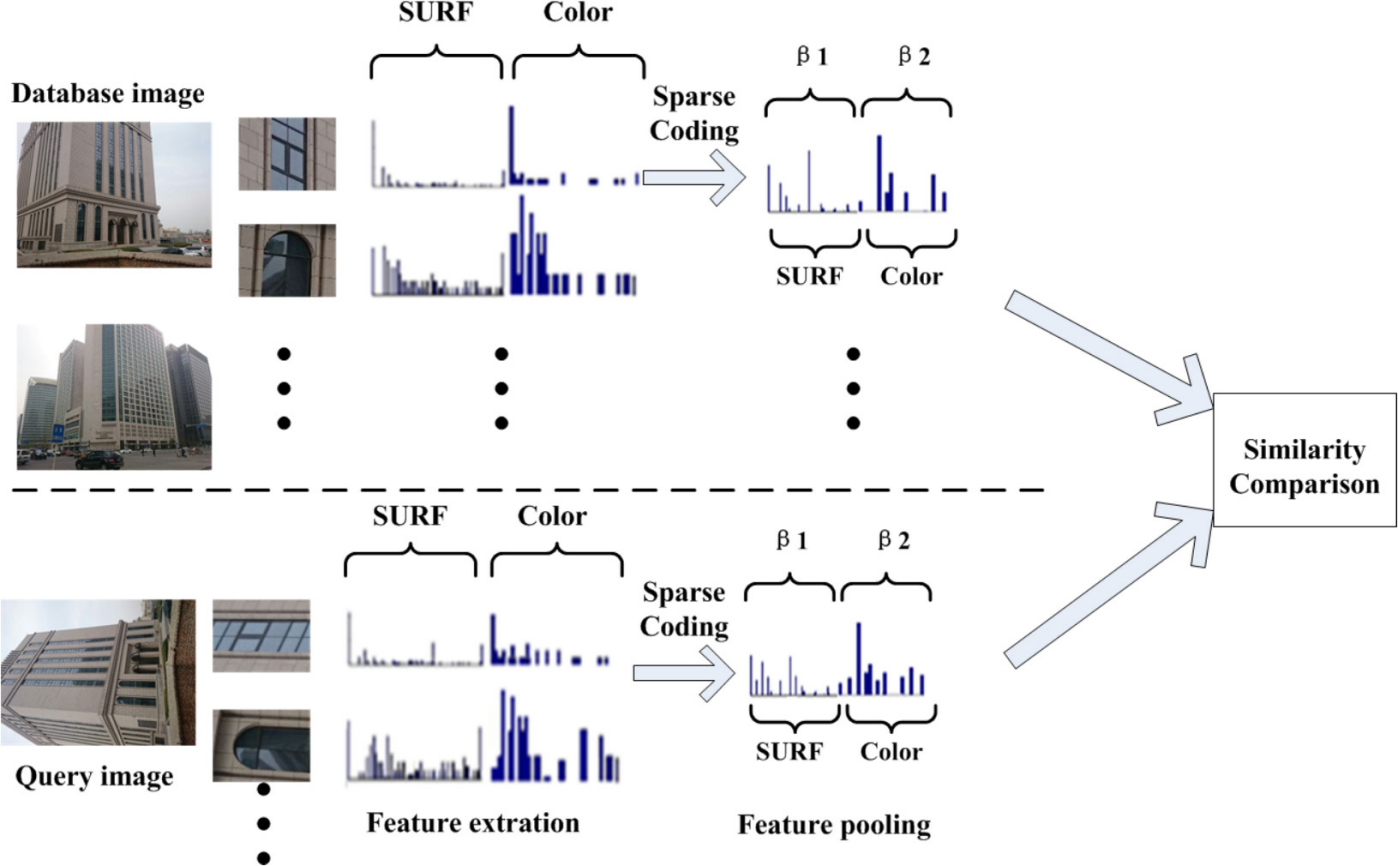
Flowchart of our multiple feature sparse coding method.

The following Fig 4 illustrates the flowchart of our multiple feature sparse coding method. The final coding vector is then obtained by applying *L*_2_-normalization. PCA, LDA or product quantization [54] can further compress the aggregated image descriptor vector into a more compact one. The similarity measure between two images can be obtained by computing the cosine distances of image representation between the query image and train images.

## Experiments

In this section, first we experimentally analyze the effects of different sampling strategies and pooling methods on image retrieval performances. Then we compare the sparse coded image retrieval method with the state-of-the-art methods, which include BoW, Fisher and VLAD. In our experiments, we set sparsity parameter *λ* = 0.15.

### Evaluation Datasets

Three common databases are used to evaluate our method. For codebook training, classical k-means method is used to cluster heterogeneous local features respectively sampled in corresponding datasets.

- **University of Kentucky Benchmark dataset (UKB)** [55]: UKB dataset contains 10200 images, which have been divided into 2550 groups. For UKB dataset, the common performance metric is defined as the average number of relevant images in the top 4 retrieval images. The images in UKB dataset are the original images with resolution of 640×480.
- **Zurich building dataset** [56]: Zurich dataset is composed of 1005 Zurich city building images and 115 query images. Similar to UKB dataset, the average number of relevant images in the top 5 retrieval images is employed as the accuracy measurement. In our experiments, each Zurich image is down sampled to 320×240.
**INRIA Holidays dataset** [57]: The INRIA Holidays dataset consists of 1491 holiday images from personal holiday photos and 500 query images. We resize the Holidays dataset images to a maximum of 786432 pixels. In this dataset, the mean Average Precision (mAP) is used to measure the retrieval accuracy.

### Different Sampling Results with Experiment Verification

We utilize Zurich and UKB datasets to evaluate the effects of sampling strategies. On each image, SURF features with a threshold 0.0001 are sparse extracted as keypoints and 16×16 image patches with 6-pixel grid spacing are used as the basic dense features. Spatial pyramid pooling (SPM) [58] is not used. After extracting those features, feature-sign method are used for encoding.

Table 1 and Table 2 demonstrate the image retrieval accuracy with different sampling strategies on both Zurich and UKB datasets respectively. As shown in Table 1, sparse sampling strategy outperforms dense sampling strategy on Zurich dataset. And the max pooling method performs significantly better than sum pooling no matter which sampling strategy is employed, especially with the increasing of vocabulary dimension. While on UKB dataset, Table 2 shows us that dense feature with max pooling strategy has the best performance. With 4*K* encoding dimension, the average recall of the top 4 ranked images is 3.3.

**Table 1:** Retrieval results with different sampling strategies on Zurich dataset.

Vocabulary dimension	Dense sampling(level = 1×1)		Sparse sampling	
	Sum	max	sum	max
512	2.2350	3.8600	3.8085	3.5740
1K	2.2720	3.9915	3.9650	**3.9925**
2K	2.2785	4.0350	4.0785	**4.1915**
3K	2.3650	4.0260	4.1130	**4.2600**
4K	2.3915	4.0260	4.2000	**4.3785**

**Table 2:** Retrieval results with different sampling strategies on UKB dataset.

Vocabulary dimension	Dense sampling(level = 1×1)		Sparse sampling	
	Sum	max	sum	Max
512	2.2510	2.7565	2.6868	2.8107
1K	2.3169	2.8504	2.7073	2.9163
2K	2.3767	**3.0990**	2.7830	3.0427
3K	2.4085	**3.2045**	2.8013	3.1102
4K	2.5178	**3.3157**	2.8501	3.1956

**Fig 5:**
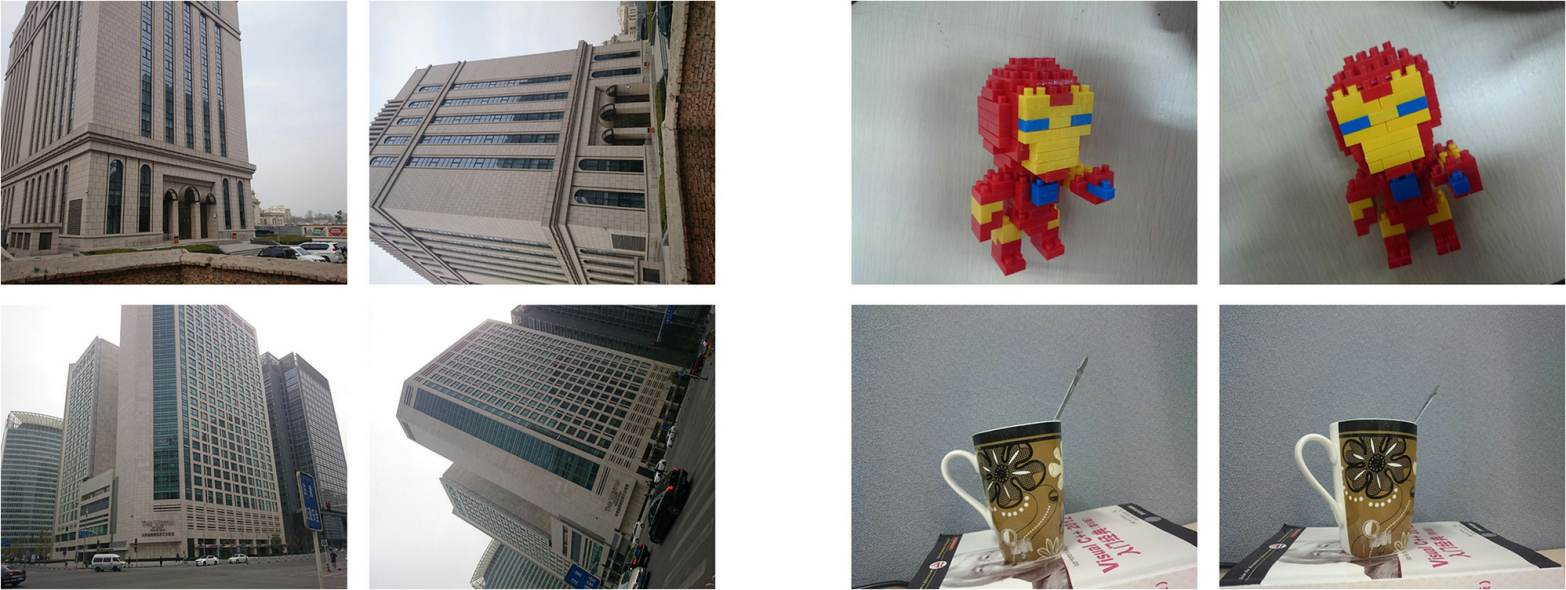
Frequent patches and distinctive patches. (a) Example images which include frequent patches on the background. (b) Example images which include distinctive patches on the background.

As mentioned in Section 3.1, patches on the background can be divided into distinctive patches and frequent patches. From the experimental results shown in Table 1 and Table 2, we can learn that for a image dataset which includes plenty of frequent patches on the background (see Fig 5A), the key-point based sampling approach can achieve better performance. When a dataset includes many distinctive patches on the background, (see Fig 5B), dense sampling strategy may provide more discriminative power.

**Fig 6:**
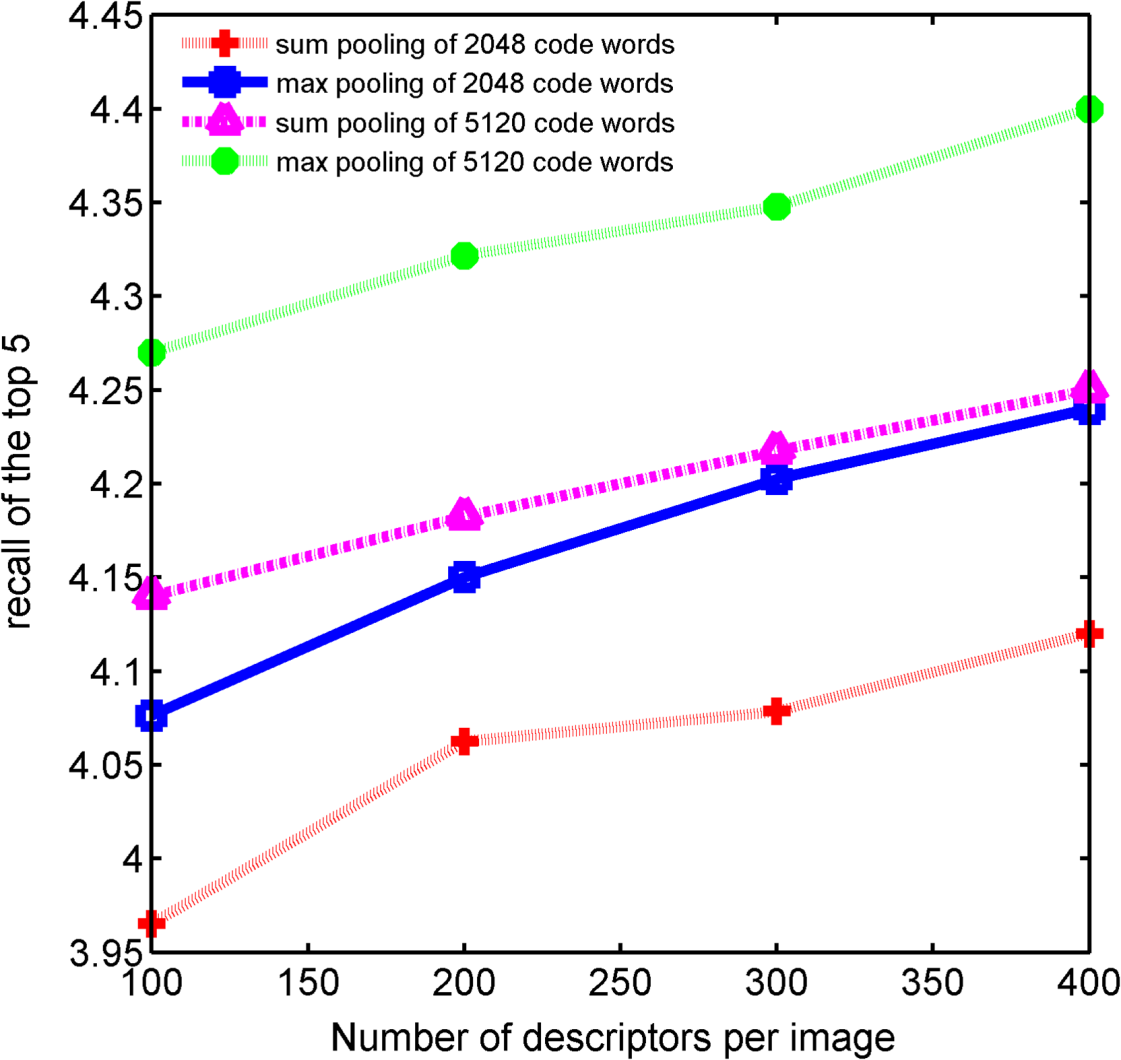
Retrieval accuracy with different pooling cardinality and number of code words.

Here we also check the effect of varying the number of SURF features extracted using key-point detector under a sparse coding framework. For key-point detectors, we simply varied the corner threshold. Fig 6 shows the average retrieval accuracy on Zurich dataset. We can see clearly that the retrieval accuracy improves as the average feature extracted on each image increases, no matter which pooling method is utilized. This experimental results support the probabilistic explanation described in section 3.2.

Therefore in following experiments, we choose to use key-point based sampling strategy to extract image features, we think it is a tradeoff between efficiency and accuracy.

### Performance of Our Proposed Pooling Method

In this section, we compare the different aggregation methods, max, sum and our proposed pooling methods, under a sparse coding framework with SURF feature only. The retrieval accuracy is also compared with state-of-the-art methods, such as BoW, VLAD and Fisher. We do not apply any post-processing procedure. For Fisher and VLAD, we use the method proposed in reference [5] directly. In Fisher a 64 SURF feature descriptor is encoded into a 2×64×*k* dimensional vector, while in VLAD the resulting dimension is 64×*k*, here *k* is the codebook size. Three datasets are utilized to evaluate the image retrieval accuracy.

Table 3 shows us the image retrieval results on Zurich dataset. As is shown, on the Zurich dataset the sparse coding method outperforms VLAD and Fisher. When features are encoded with 8*K* visual codes and max pooled, about a 7% improvement is observed. Our modified pooling strategy outperforms max pooling when coding dimension is below 4*K*. The retrieval result is just 3.85 when BoW framework with 10^6^ visual words is applied on Zurich dataset.

**Table 3:** Retrieval results with different aggregation methods with single SURF feature on Zurich dataset.

Approaches		1K	2K	4K	8K
Fisher		3.6260	3.9045	4.0955	4.1045
Vlad		3.7610	3.8955	4.0435	4.1130
SC	Sum	3.9650	4.0785	4.2000	4.2955
	max	4.0125	4.1915	4.3085	**4.4435**
	**Our**	4.1390	4.2435	4.2870	4.3940

Table 4 illustrates that on the UKB dataset we can achieve 3.35 score with max pooling, which significantly exceeds the Fisher and VLAD by 5%. On the UKB dataset, our pooling method can improve the retrieval results from 2.78 to 3.17, when coding dimension is as low as 2*K*. When a BoW model with 10^6 codebook is employed, the result is 2.75 of the recall of top 4 on UKB dataset.

**Table 4:** Retrieval results with different aggregation methods with single SURF feature on UKB dataset.

Approaches		1K	2K	4K	8K
Fisher		2.7784	2.8210	2.9832	3.2143
VLAD		2.7312	2.8237	2.9526	3.2002
SC	Sum	2.7573	2.7830	2.9501	3.0112
	max	3.0055	3.1102	3.2956	**3.3508**
	**Our**	3.0736	3.1720	3.3091	3.3380

For Holidays dataset, the results shown in Table 5 demonstrate that the retrieval accuracy is 0.67 using our proposed method.

**Table 5:** Retrieval results with different aggregation methods with single SURF feature on Holidays dataset.

Approaches		1K	2K	4K	8K
Fisher		0.5773	0.6327	0.6373	0.6579
VLAD		0.5659	0.6204	0.6371	0.6541
SC	Sum	0.5459	0.5798	0.5969	0.6140
	Max	0.5965	0.6271	0.6435	**0.6757**
	**Our**	0.6057	0.6334	0.6572	0.6700

Above results show that alleviating the higher weights of some visual words, which most probably are the burst visual features, will help to improve the retrieval performance. Moreover, eliminating smaller coding coefficients, which may be only assigned by one feature, will improve retrieval accuracy further.

## Sparse Coding with Multiple Features

**Fig 7:**
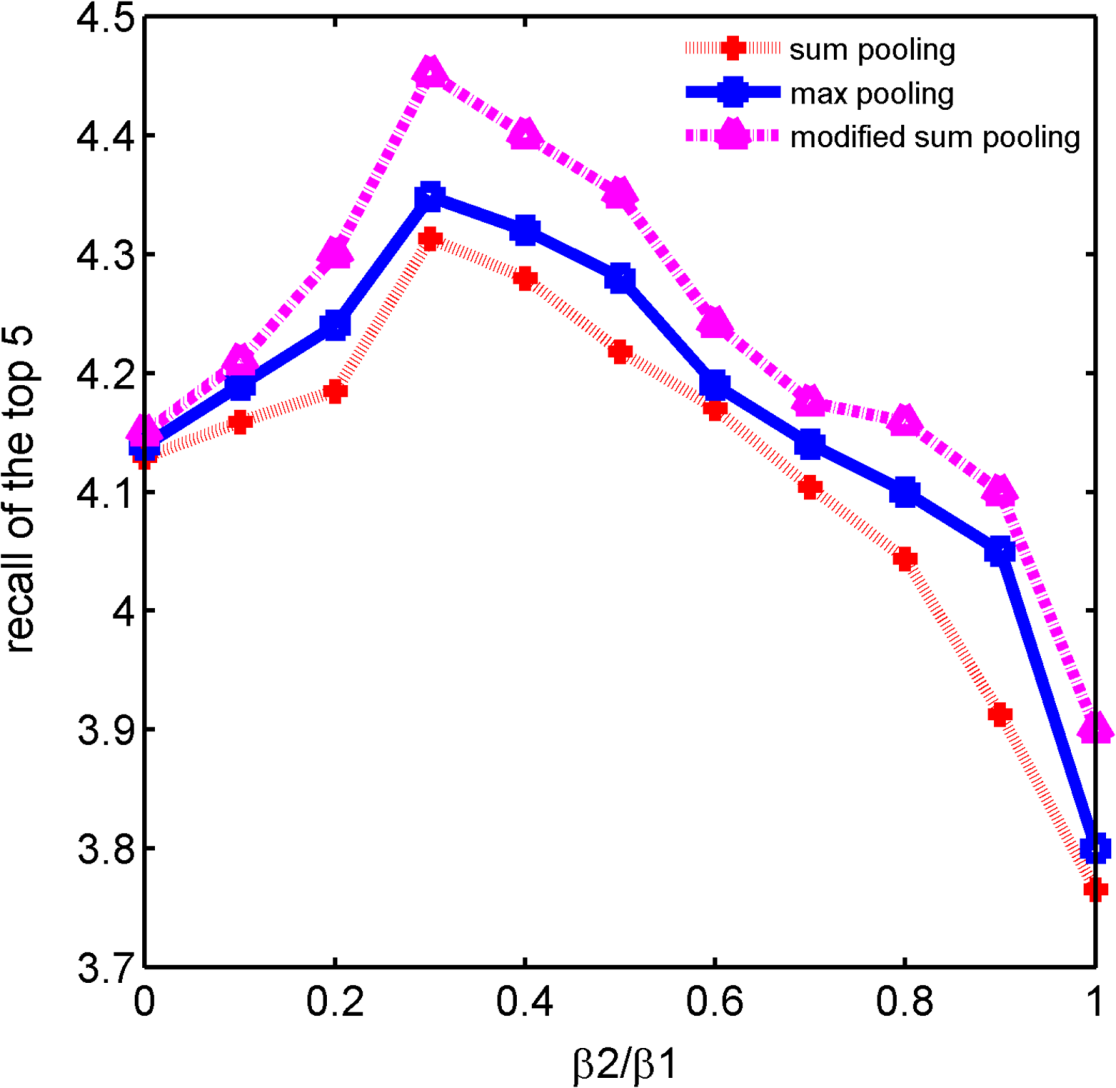
Impact on retrieval accuracy with different combining parameters for SURF and color features.

In this experiment we combine SURF feature with opponent color feature together under a sparse coding framework. We fix the color codebook size as 1*K* and change the SURF codebook size. Fig 7 described how the weight parameters (*β*_1_,*β*_2_) affect the retrieval accuracy in Zurich dataset with a 5*K* visual codebook. The parameters we used are approximate for UKB and Holidays datasets. Therefore in our experiments we choose *β*_2_ / *β*_1_ = 0.3 as an optimal weight ratio. Tables 6, 7 and 8 show image retrieval accuracy results with multiple features under a sparse coding framework on three datasets respectively.

**Table 6:** Retrieval results with multiple features on Zurich dataset.

Dimension(SURF+color)	2K	3K	5K	9K
Sum	4.0384	4.2027	4.3130	4.4350
Max	4.1826	4.2783	4.3480	4.4955
**Our**	4.2630	4.3825	4.4520	**4.5380**

**Table 7:** Retrieval results with multiple features on UKB dataset.

Dimension(SURF+color)	2K	3K	5K	9K
Sum	3.3675	3.3983	3.4127	3.4176
Max	3.4120	3.5020	3.6128	3.7135
**Our**	3.4647	3.5210	3.6385	**3.7278**

**Table 8:** Retrieval results with multiple features on Holidays dataset.

Dimension(SURF+color)	2K	3K	5K	9K
Sum	0.6700	0.6833	0.7046	0.7351
Max	0.7008	0.7280	0.7458	0.7539
**Our**	0.7194	0.7351	0.7595	**0.7651**

As the above results demonstrated to us, multiple descriptors can bring a significant improvement over three datasets. Particularly with our modified pooling method, the retrieval accuracy can outperform max pooling method both in low dimension and high dimension. It can achieve 4.54 on Zurich dataset of the recall of top 5, 3.73 on UKB dataset of the recall of top 4 and a mAP of 0.76 on Holidays dataset. As verified in section 5.3, our modified pooling strategy outperforms max pooling when the codebook size is below 4*K*. Though the SURF coding dimension is high, the color codebook size is just within this range. Therefore, because of the contribution of color features, our pooling method outperforms max pooling.

Compared with the results in [14], we can see that opponent color features extracted around key-point rather than micro dense sampling, combined with SURF feature can also be a good feature combination. The retrieval results are approximate but the time consumed is less.

## Conclusions

Sparse coding scheme can encode feature descriptors from an image into a fixed size image vector, which has been successfully used in image classification. However, using sparse coding scheme for image retrieval has not been intensively studied. In this paper, we have not only analyzed the effects of feature extraction and pooling strategies on image retrieval performance under sparse coding framework, but also aggregated SUFR and color descriptors together for large-scale image retrieval. By further incorporating color feature, our sparse coding scheme achieves better performance on several benchmark databases than the-state-of-art methods. Moreover we have discussed the probabilistic essence of sum and max pooling and proposed a modified sum pooling strategy which can improve the retrieval accuracy significantly, especially for smaller visual vocabularies. In the future, more efforts will be made to explore the intrinsic properties of max pooling and to reduce the computation complexity of sparse coding method.
